# Jiwar: A database and calculator for word neighborhood measures in 40 languages

**DOI:** 10.3758/s13428-025-02612-7

**Published:** 2025-02-19

**Authors:** Alaa Alzahrani

**Affiliations:** Riyadh, Saudi Arabia

**Keywords:** Neighborhood database, Neighborhood calculator, Orthographic neighborhood, Phonological neighborhood, Phonographic neighborhood, Multilingual

## Abstract

The majority of neighborhood calculators are restricted to one language. The limited availability of multilingual neighborhood calculators could pose challenges for conducting psycholinguistic research on low-resource languages. Therefore, this study introduced Jiwar, a database and calculator for neighborhood information across three levels (orthographic, phonological, and phonographic) across 40 languages. The database contains information for 24 linguistic and neighborhood measures, while the Python-based calculator allows users to compute more than 46 neighborhood measures for words and nonwords. This study further examined the Jiwar calculator’s instrument reliability and validity. Correlations with previous datasets across several languages suggested the strong reliability of two key Jiwar measures. Multiple-linear regression models revealed that a subset of Jiwar measures significantly predicted behavioral results in lexical decision and visual naming tasks, indicating the validity of the Jiwar calculator. Jiwar is an open-source, Python-based tool that is designed to expand to more languages and functions.

## Introduction

In the psycholinguistic literature, the relationship between words is studied using the concept of neighborhood. A word’s neighborhood refers to the set of words that are identical to the target word except for a single letter (orthography), phoneme (phonology) or both (phonography). For instance, the word “stove” exhibits orthographic similarity with “stone” and “shove” since they differ by a single letter from the target word. Meanwhile, “stove” displays phonological similarity with “stone” and “stow” as they differ by a single phoneme from the target word. Words that demonstrate both orthographic and phonological similarity to a target word, such as “stone” in relation to “stove,” are considered phonographically similar. These similar words are known as “neighbors” and have been found to significantly influence lexical processing (for reviews, Perea, 2015; Perea & Rosa Martínez, 2000; Vitevitch & Luce, 2016). The influence of word neighbors is known as the neighborhood effect. The impact of word neighbors has been observed across different experimental paradigms, including eye-tracking during reading (e.g., Johnson, 2009), lexical decision tasks (e.g., Ziegler et al., 2003), naming tasks (e.g., Siew, 2017), lexical priming tasks (e.g., Van Heuven et al., 2001), and event-related potentials (e.g., Midgley et al., 2008). Although the neighborhood effect is robust, current evidence suggests that its direction and strength may differ across languages (e.g., Arutiunian & Lopukhina, 2020; Sadat et al., 2014; Vitevitch & Rodríguez, 2005).

Despite this cross-linguistic variation in neighborhood effects, the available neighborhood calculators in the literature tend to be restricted to one language such as English (Davis, 2005; Storkel & Hoover, 2010), Spanish (Davis & Perea, 2005) Persian (Esmaeelpour et al., 2022), Arabic (Aljasser & Vitevitch, 2022), and Basque (Perea et al., 2006), hindering the examination of neighborhood effects across several languages. Meanwhile, the few available multilingual neighborhood calculators require the preparation of a custom database for computing neighborhood measures (Chee et al., 2021; Westbury et al., 2007), a step that can be challenging, particularly for low-resourced languages. The limited availability of multilingual neighborhood databases and automated calculators could prevent us from gaining important insights about lexical processing in typologically unique languages. This gap could further contribute to marginalization of less-explored languages and the overreliance on Western, Educated, Industrialized, Rich and Democratic (WEIRD) samples in psycholinguistics and bilingual research (Andringa & Godfroid, 2020; Blasi et al., 2022; Garcia et al., 2022; Jaeger & Norcliffe, 2009; Kidd & Garcia, 2022; Plonsky, 2023). To address this lack, the current study developed and validated a multilingual neighborhood calculator called Jiwar (an Arabic term meaning “neighboring”). Jiwar functions as both a neighborhood database and a computational tool for generating neighborhood measures across three dimensions: orthographic, phonological and phonographic. In the following, an overview of the relevant literature is presented followed by the introduction and validation of the Jiwar calculator.

## Literature review

### Neighborhood measures

A word's orthographic, phonological, or phonographic neighborhood can be measured using several methods. Early research on neighborhood effects primarily focused on orthographic neighborhoods (Coltheart et al., 1977), leading to the development of measures that initially targeted only orthographic neighbors. Subsequently, these measures were adapted to account for phonological (e.g., Suárez et al., 2011) and phonographic neighbors (e.g., Costa et al., 2023). Therefore, in this section, we will discuss key orthographic neighborhood measures and briefly outline their phonological and phonographic equivalent measures. This discussion will focus on measures commonly used in research on neighborhood effects (Perea, 2015; Vitevitch & Luce, 2016), as well as those found in neighborhood databases (Costa et al., 2023; Guasch et al., 2013; Marian et al., 2012) and calculators (Chee et al., 2021; Davis, 2005; Westbury et al., 2007).

#### Orthographic neighborhood measures

A classic definition of orthographic similarity considers words as neighbors if they differ by only one letter while maintaining the identity and position of other letters (Coltheart et al., 1977). For example, “cat” has orthographic neighbors such as “oat,” “cot,” “vat,” “cab,” and “mat.” Based on this definition, orthographic neighborhood size has been operationalized as the number of neighbors differing by a single letter substitution, either in the same position (e.g., Balota et al., 2007; Yap et al., 2010) or in any position (e.g., Keuleers et al., 2010, 2012). It has been suggested that the position of the substituted letter may minimally influence written word recognition, while the substitution of a letter, whether at the beginning or end of a word, could exhibit more influence on spoken word recognition (De Cara & Goswami, 2002; Vitevitch, 2002). The number of word neighbors is referred to by various terms in the psycholinguistic literature, including neighborhood density (e.g., Ktori et al., 2008), neighborhood size (e.g., Nemati et al., 2022), Coltheart’s N (e.g., Yarkoni et al., 2008), or simply N (e.g., Parker et al., 2021). Words with numerous neighbors (e.g., “pale “, *N* = 20) are described as having large or dense neighborhoods, while those with few neighbors (e.g., “trek”, *N* = 1) are characterized as having small or sparse neighborhoods (e.g., Baus et al., 2008).

The classic definition of orthographic similarity, which focuses solely on substitution-letter neighbors, has been criticized for two key limitations. First, this restrictive definition overlooks other types of neighbors, such as addition-letter (e.g., “stand” for “sand”) and deletion-letter (e.g., “sad” for “sand”) neighbors, which have been found to influence lexical processing (e.g., Davis & Bowers, 2006; Davis & Taft, 2005; Davis et al., 2009; Duñabeitia & Vidal-Abarca, 2008; Lavidor et al., 2006). Second, the classic definition does now allow the calculation of orthographic neighborhood size for a given word that differs from other words in the lexicon by two or more letters (Suárez et al., 2011). Such words constitute 2.5% of monosyllabic and 43.9% of disyllabic words in the English Lexicon Project (Balota et al., 2007).

In response to these shortcomings, recent research has adopted a broader definition of orthographic neighborhood similarity that includes neighbors differing by a single letter through substitution, addition, or deletion (e.g., Ferrand et al., 2010). In this study, we will use the term orthographic neighborhood density (OND) to refer to this definition. Additionally, a new expansive measure of neighborhood size, orthographic Levenshtein distance 20 (OLD20), has been proposed (Yarkoni et al., 2008). This measure is based on the Levenshtein distance between two words. The Levenshtein distance refers to the minimum number of single-letter changes (substitution, addition, or deletion) needed to change one word to the other. For example, the Levenshtein distance between “sand” and “sandy” is 1 because only one letter (i.e., “y”) was added. The OLD20 measure is defined as the mean Levenshtein distance between a target word and its closest 20 words in the lexicon. Strengths of OLD20 include its powerful predictive ability for longer words, its capability to handle words with minimal to no neighbors (i.e., hermits), and its stronger sensitivity of neighborhood frequency effects compared to Coltheart’s N (Suárez et al., 2011; Yarkoni et al., 2008). More information about hermit words can be found in Vitevitch (2008) and Suárez et al. (2011). OLD20 has become one of the most employed neighborhood measures in visual word recognition research (Perea, 2015).

However, both OND and OLD20 measures do not take into account the mediating role of orthographic neighbor frequency. There is some evidence that words with higher-frequency orthographic neighbors show different effects than those with lower-frequency orthographic neighbors (Grainger & Segui, 1990). To work around this issue, existing studies have employed additional measures to quantify the effects of neighbor frequency. Most studies calculate the mean frequency of all neighboring words (e.g., Aithal et al., 2024; Alexeeva et al., 2018; Luce & Pisoni, 1998; Vitevitch & Rodríguez, 2005), whereas some studies calculate the mean frequency of neighbors with frequencies higher or lower than the target word (e.g., Hameau et al., 2021b; Martín & Pérez, 2008).

While the measures discussed previously focus on the relationship between target words and their neighbors, recent research has highlighted the importance of relationships among the neighbors themselves (e.g., Chan & Vitevitch, 2009, 2010; Vitevitch et al., 2012). The clustering coefficient (C) is a common measure used to calculate the similarity relationships among neighbors of a given word (e.g., Brown et al., 2018; Siew, 2017, 2018). This measure is based on the network science method commonly used in the cognitive science field (Baronchelli et al., 2013; Siew et al., 2019). The network method has been developed to study complex systems that involve multiple components interacting with one another, resulting in complex behavior. Network science has subsequently been introduced to psycholinguistic research to examine orthographic or phonological similarity between words (Chan & Vitevitch, 2009; Siew, 2018). This network approach enables researchers to model the complex connections between words within the mental lexicon. In this approach, words are represented as nodes, and links between nodes signify orthographic or phonological similarities. The clustering coefficient C measure is typically calculated using the Pajek computer program (Batagelj & Mrvar, 1988) in psycholinguistic studies (e.g., Chan & Vitevitch, 2009, 2010; Vitevitch et al., 2012). The clustering coefficient (C) quantifies the degree of interconnectedness among the neighbors of a given target word. It is calculated as the ratio of the actual number of existing links between these neighbors to the number of all possible links that could exist if all neighbors were connected to each other (Batagelj & Mrvar, 1988). The mathematical formula used to calculate the clustering coefficient is as follows (Vitevitch et al., 2011):

$$\frac{\text{C}{\text{i}} = 2 |\text{e}{\text{jk}}| }{(\text{k}{\text{i}}(\text{k}{\text{i}} \, - 1))}$$

In this formula, i refers to a given word, while e_jk_ represents the presence of connections between the neighbors j and k of word i. The vertical lines |…| indicates that we count the number of these connections. K_i_ is the degree (neighborhood density) of the given word i, that is how many neighbors it has. As such, the clustering coefficient C refers to the number of actual connections among the neighbors of a given word divided by the maximum number of connections that could possibly exist among the neighbors of that word. In terms of orthographic neighbors, the orthographic clustering coefficient C refers to the proportion of orthographic neighbors of a target word that are also orthographic neighbors of each other.

#### Phonological neighborhood measures

Phonological neighborhood measures are analogous to the above-discussed orthographic measures, with the exception that they focus on single-phoneme edits rather than single-letter edits. For instance, phonological neighborhood density refers to the number of words in the lexicon that differ from a target word by one phoneme, either via substitution, addition, or deletion (Luce, 1987; Luce & Pisoni, 1998). Although this one-phoneme metric is widely used, it should be noted that phonological similarity between words may be defined in different ways, as described in Luce and Pisoni (1998) as well Castro and Vitevitch (2023). Another known measure of the phonological neighborhood is the phonological Levenshtein distance 20 (PLD20). This measure is the phonological equivalent of OLD20, and it calculates the mean phonological Levenshtein distance to the 20 closest phonologically similar words in the lexicon. Furthermore, the phonological clustering coefficient C refers to the degree to which a word's phonological neighbors are also phonological neighbors of each other.

Furthermore, a recently introduced measure of phonological neighborhood is 2-hop density (Siew, 2017). Like the clustering coefficient C, 2-hop density is a measure based on network science. 2-hop density measures how interconnected a word's distant phonological neighbors are with its immediate neighbors. In network science research, immediate neighbors (1-hop) are words that can be transformed into the target word by substituting, adding, or deleting one phoneme in any position. Distant neighbors (2-hop) are words that are indirectly connected to the target word via these immediate neighbors; they are direct neighbors of the target word's immediate neighbors, but not of the target word itself. For example, for the word “cat”, words like “cut” and “at” are 1-hop neighbors because they differ by a single phoneme. Meanwhile, words like “cup” and “ant” are 2-hop neighbors because they are indirectly connected to “cat” via its immediate neighbors “cut” and “at” respectively. 2-hop density is calculated as the ratio of actual connections between 1-hop and 2-hop neighbors to all possible connections that could exist between them. Siew (2017) reported that English words found in less interconnected 2-hop neighborhoods (low 2-hop density) were named and responded to more quickly than English words found in more interconnected 2-hop neighborhoods (high 2-hop density). This evidence indicates that the connectivity of distant phonological neighbors also impacts speech production and recognition.

#### Phonographic neighborhood measures

Phonographic similarity could be an important factor in lexical processing especially in languages such as English, Arabic, and Hebrew, where spelling and pronunciation do not always coincide (Frost, 2012). In this case, words with many phonographic neighbors may elicit different behavioral results than words with fewer phonographic neighbors, potentially triggering a facilitatory phonographic neighborhood effect on word processing. Specifically, words with many phonographic neighbors were named and recognized more quickly than words with fewer phonographic neighbors in visual word recognition tasks (speeded naming, visual lexical decision) (Siew & Vitevitch, 2019).

Phonographic neighborhood measures are comparable to the above-outlined orthographic measures except that they focus on single-phoneme and single-letter edits rather than single-letter edits. Phonographic neighborhood density relates to the number of words that differ from a target word by one phoneme and one letter via substitution, addition, or deletion. For example, the word “cat” (/kæt/) has phonographic neighbors such as “bat” (/bæt/), “cats” (/kæts/), and “cap” (/kæp/). Meanwhile, the phonographic Levenshtein distance 20 (POLD20), inspired by OLD20, estimates the mean Levenshtein distance between a target word and its 20 closest phonographically similar words in the lexicon. Finally, the phonographic clustering coefficient C estimates the degree to which a word's phonographic neighbors are also phonographic neighbors of each other.

### Neighborhood effects

A large body of research has demonstrated that the orthographic, phonological, and phonographic neighbors of a word significantly influence various aspects of language processing, such as word recognition, production, and acquisition. Crucially, the direction of these neighborhood effects varies across languages, highlighting the need to consider language-specific characteristics. The following sections will provide an overview of empirical findings from neighborhood effect studies, emphasizing cross-linguistic variability to underscore the need for a multilingual neighborhood database and calculator.

#### Orthographic neighborhood studies

Orthographic neighbors have been shown to affect distinct dimensions of language processing (recognition, production) across different task modalities (visual, auditory) and speaker groups (adults, children). In visual word recognition tasks (e.g., visual lexical decision, visual naming), both children and adults recognize words with many orthographic neighbors significantly faster and more accurately than words with few neighbors (Duñabeitia & Vidal-Abarca, 2008; Laxon et al., 1988; for a review, Perea & Rosa Martínez, 2000). However, the finding that words with many neighbors are recognized/read more quickly than those with few neighbors appears to be modulated by language-specific features such as the writing script (Huang et al., 2006; Nemati et al., 2022) and word structure (Ziegler & Perry, 1998). In auditory word recognition (e.g., auditory lexical decision), words belonging to larger orthographic neighborhoods have also been found to elicit faster and more accurate responses (Vitevitch & Rodríguez, 2005; Ziegler et al., 2003). Orthographic neighbors further impact word production. In written word production tasks, a robust body of research has consistently demonstrated that children exhibit enhanced spelling accuracy for words with numerous substitution neighbors in the initial letter position (e.g., cat, mat, sat) (Chen et al., 2021; Deavers & Brown, 1997; Goswami & Mead, 1992). In spoken word production tasks, orthographic neighbors could influence the accuracy of word pronunciation (Kucker & Perry, 2023).

#### Phonological neighborhood studies

Phonological neighbors have been reported to influence different aspects of language processing (recognition, production, acquisition) across task modalities (visual, auditory) and speaker groups (children, adults, and sign language users). In visual word recognition, it is consistently reported that phonological neighborhood density facilitates visual word recognition. Words with many phonological neighbors are responded to more rapidly and accurately compared to words with few phonological neighbors (Yates, 2005; Yates et al., 2020). In auditory word recognition tasks (e.g., auditory lexical decision), research has revealed a significant effect of phonological neighborhood size on word recognition for children and adults (e.g., Garlock et al., 2001; Goh et al., 2009). However, the direction of this effect differs across languages (for a review, Vitevitch & Luce, 2016). For instance, the effect of phonological neighborhood density is typically inhibitive in English and French, with reduced speed and accuracy for recognizing words with larger phonological neighborhoods (Dufour & Frauenfelder, 2010; Goh et al., 2009; Luce & Pisoni, 1998; Vitevitch & Luce, 1998). However, one recent French study found the opposite pattern, with faster responses for words that are phonologically similar to many other words than those with few phonological neighbors (Ferrand et al., 2018). Meanwhile, words with a higher number of phonological neighbors are recognized faster than words with sparse phonological neighborhoods in Spanish (Vitevitch & Rodríguez, 2005) and Russian (Arutiunian & Lopukhina, 2020). On the other hand, in Chinese, the effect is graded; distant phonological neighbors increase the speed of word recognition, while near phonological neighbors reduce the speed of word recognition (Li et al., 2024). Based on a similarity judgement task, Li et al. found that native Mandarin speakers perceived words with a single constitute-edit as the most dissimilar (/hwan1/, /kan1/), while those differing by a single tone-edit (/hwan1/, /hwan2/) or a single phoneme-edit (/hwan1/, /wan1/) were viewed as similar. The authors termed the highly dissimilar pairs “distant neighbors”, and the similar pairs “near neighbors”.

A similar pattern emerges from spoken word production studies. English studies have shown that words with a dense phonological neighborhood are more likely to be produced accurately and rapidly than those with a sparse neighborhood (Stemberger, 2004; Vitevitch, 1997; Vitevitch & Sommers, 2003), with possible frequency effects (Hameau et al., 2021b). On the other hand, research on native speakers of Spanish (Sadat et al., 2014; Vitevitch & Rodríguez, 2005) and Russian (Arutiunian & Lopukhina, 2020) showed the opposite pattern of results: slower production for words with dense neighborhoods than words with sparse neighborhoods. Phonological neighborhood size has further been found to influence the explicit and implicit acquisition of vocabulary (James et al., 2021; Storkel, 2009; Storkel & Lee, 2011; Storkel et al., 2006; van der Kleij et al., 2016), bilingual word processing (Botezatu & Garcia, 2024; Hameau et al., 2021a, 2021b) and lexical recognition in sign language (Caselli et al., 2021).

#### Phonographic neighborhood studies

The effects of phonographic neighbors have been examined in two task modalities. In visual word recognition tasks, words with more phonographic neighbors are named faster than those with fewer phonographic neighbors (Adelman & Brown, 2007; Peereman & Content, 1997). In spoken word recognition tasks, words with higher phonographic C were more rapidly recognized than words with lower phonographic C (Siew & Vitevitch, 2019). These findings, while promising, offer preliminary evidence based on a small subset of languages, including English and French.

#### Neighborhood effects on nonwords

Nonwords (also known as pseudowords) are combinations of letters that conform to the orthographic rules and phonotactic constraints of a given language while lacking meaning (Thomson et al., 2006). Research has shown that orthographic neighborhood size influences nonword reading performance. For example, the English nonword “dast” has multiple orthographic neighbors (including “dust,” “dash,” “dart,” and “daft”), while “toosh” has only one neighbor (“tooth”) (Siakaluk et al., 2002). Studies in English, Dutch, and Italian have demonstrated that nonwords with many orthographically similar words are read aloud faster and more accurately than nonwords with few orthographic neighbors (e.g., Arduino & Burani, 2004; Janse & Newman, 2013; Laxon et al., 2002; McCann & Besner, 1987). However, these findings come primarily from a small number of dominant Indo-European languages. Since neighborhood effects on the processing of words tended to vary by the examined language (see discussion above), it is possible that a similar cross-linguistic variation would be observed for the processing of nonword (e.g., Goswami et al., 2001). Given this influence, researchers may need to measure the neighborhood size of nonwords to control the experiment and ensure that the nonwords match the linguistic characteristics (length, neighborhood size, etc.) of the real words used in a lexical decision task.

### Neighborhood databases

A large number of orthographic and/or phonological neighborhood databases are found in the literature. An overview of key databases is presented in Table 1. While these databases have proved invaluable and have advanced language processing research, they nonetheless exhibit several limitations. First, they predominantly cover a small subset of Indo-European languages, resulting in poor resources for less-investigated languages. This gap could perpetuate the overrepresentation of well-studied languages in psycholinguistic research (e.g., Andringa & Godfroid, 2020; Blasi et al., 2022; Garcia et al., 2022; Kidd & Garcia, 2022) while neglecting less-investigated languages. Second, many databases lack comprehensive neighborhood measures across orthographic, phonological, and phonographic domains. The availability of a restricted range of neighborhood measures could pose challenges for a thorough understanding of the often multi-dimensional neighborhood effects on lexical processing (e.g., Adelman & Brown, 2007; Peereman & Content, 1997; Siew & Vitevitch, 2019). Third, some databases omit critical frequency information for word neighbors, hindering research on the well-established interaction between neighborhood effects and word frequency (e.g., Hameau et al., 2021a, 2021b). Fourth, while some languages have large databases (range = 5.5 billion to 5–100 million words), others have a relatively small number of lexical entries (range = 1152 to 14,365 words), thus leaving a substantial portion of the language's lexicon unexplored. These shortcomings in existing databases may impede research on lexical processing, particularly for understudied languages, which often lack readily available materials and resources.

**Table 1:** A summary of orthographic and phonological neighborhood databases across languages and their features

Name	Reference	N Lg	Language family	Multi	Orth	Phon	Phonog	WF	NF	Word
PHOR-in-One	Costa et al. (2023)	4	Indo-European	✓	✓	✓	✓	✓		6160
CLEARPOND	Marian et al. (2012)	5	Indo-European	✓	✓	✓		✓	✓	27,751
NIM	Guasch et al. (2013)	3	Indo-European	✓	✓			✓		5–100 million
Persian Lexicon Project	Nemati et al. (2022)	1	Indo-European		✓			✓		64,546
English Lexicon Project	Balota et al. (2007)	1	Indo-European		✓	✓		✓	✓	40,481
British Lexicon Project	Keuleers et al. (2012)	1	Indo-European		✓			✓		14,365
French Lexicon Project	Ferrand et al. (2010)	1	Indo-European		✓			✓		38,840
Chronolex	Ferrand et al. (2011)	1	Indo-European		✓	✓		✓		1482
HeLP	Stein et al. (2024)	1	Semitic		✓			✓		10,000
Malay Lexicon Project	Yap et al. (2010)	1	Austronesian		✓	✓		✓		9592
NS	Keuleers et al. (2010)	1	Indo-European		✓			✓		14,000
Developmental Lexicon Project	Schröter and Schroeder (2017)	1	Indo-European		✓			✓		1152
P-PAL	Soares et al. (2018)	1	Indo-European		✓	✓		✓	✓	208,000
childLex	Schroeder et al. (2015)	1	Indo-European		✓			✓		10 million
GreekLex	Ktori et al. (2008)	1	Indo-European		✓			✓	✓	35,304
StimulStat	Alexeeva et al. (2018)	1	Indo-European		✓	✓		✓	✓	1.7 million
LASTU	Itkonen et al. (2024)	1	Uralic		✓			✓		5.5 billion
KannadaLex	Aithal et al. (2024)	1	Dravidian			✓		✓		170,000
ONESC	Martín and Pérez (2008)	1	Indo-European		✓			✓	✓	100,944
Jiwar		40	Indo-European, Austronesian, Semitic, Turkic	✓	✓	✓	✓	✓	✓	30,000

*N Lg*, Number of Languages; *Multi*, Multilingual; *Orth*, Orthographic measures; *Phon*, Phonological measures; *Phonog*, Phonographic measures; *WF*, Word Frequency; *NF*, Neighborhood Frequency; *Word*, Word form count. NS = the name was not specified by the authors

### Neighborhood calculators

A small number of neighborhood calculators are available in the literature. A summary of these calculators is presented in Table 2. These calculators have provided essential neighborhood information and facilitated psycholinguistic research. However, most of them are designed to generate measures for a single language only rather than multiple languages. Meanwhile, the few multilingual options lack four important features. These features include built-in corpora, support for several writing scripts, an IPA generation feature, and sufficient validation. The available multilingual calculators do not offer built-in corpora, instead requiring users to upload both a reference corpus and a word list to generate measures. While this approach offers flexibility, it may present challenges, especially for languages with limited linguistic resources. Additionally, current multilingual calculators tend to support Latin-script languages, overlooking those with different writing systems such as abjads (e.g., Arabic, Hebrew). Research has demonstrated that the language’s writing system likely influences the manifestation of neighborhood effects (e.g., Nemati et al., 2022; Stein et al., 2024). Further, existing multilingual calculators typically lack IPA transcription functionality, impeding the quick extraction of phonological and phonographic neighborhood data. Lastly, most neighborhood calculators have not been sufficiently validated, with some studies focusing solely on instrument reliability or instrument validity but not both (see the Validation section for details). However, examining both aspects of validity is essential to robustly establish the reliability and validity of any newly developed calculator (Linders & Louwerse, 2023).

**Table 2:** A summary of lexical neighborhood calculators and their features

Name	Reference	Script (script type)	N Lg	Multi	Orth	Phon	Phonog	Built-in Corpus	User corpus	IPA	Rel	Val
NS	Storkel & Hoover (2010)	Latin (Alphabetic)	1			✓		✓				✓
N-Watch	Davis (2005)	Latin (Alphabetic)	1		✓			✓	✓			
BuscaPalabras	Davis and Perea (2005)	Latin (Alphabetic)	1		✓			✓	✓			
E-Hitz	Perea et al. (2006)	Latin (Alphabetic)	1		✓	✓		✓				
NS	Aljasser and Vitevitch (2022)	Arabic (Abjad)	1			✓		✓				
WordPars	Esmaeelpour et al. (2022)	Arabic (Abjad)	1		✓	✓		✓				
LINGUA	Westbury et al. (2007)	All scripts (Alphabetic, Abjad, Abugida, Syllabary, Logographic)	*	✓	✓				✓			
LexiCAL	Chee et al. (2021)	Latin (Alphabetic)	*	✓	✓	✓	✓		✓		✓	
Jiwar		Latin, Arabic, Cyrillic, Greek, Armenian, Hangul (Alphabetic, Abjad)	40	✓	✓	✓	✓	✓	✓	✓	✓	✓

*N Lg*, Number of Languages; *Multi*, Multilingual; *Orth*, Orthographic measures; *Phon*, Phonological measures; *Phonog*, Phonographic measures; *Rel*, Reliability; *Val*, Validity. The asterisk (*) indicates that no specific number of languages was provided by the authors. NS = the name was not specified by the authors

### The current study

To address the identified limitations in the literature, this study has two objectives. First, it seeks to introduce a comprehensive, open-source database as well as a calculator that each provides three key dimensions of word neighborhood information – orthographic, phonological, and phonographic – along with corpus frequency data across 40 languages. Second, it aims to validate the introduced calculator by examining two critical aspects of validity: instrument reliability and instrument validity (see the Validation section for details).

## Method

### Jiwar

Jiwar is both a database and a calculator that generates linguistic and neighborhood measures for 40 languages. The calculator can be used to generate metrics for words and nonwords. Below, we describe Jiwar's corpus, supported languages, database features, and calculated measures.

#### Corpus

We utilized OpenSubtitles corpora for 55 languages provided by the Subs2vec Python library (Van Paridon & Thompson, 2021). This corpus was selected for its broad multilingual coverage, and its use of TV/movie subtitles, which provide superior frequency estimates compared to book-based sources (Brysbaert & New, 2009). We excluded 14 language varieties due to low correlations between their automatic IPA generation and human-generated IPA (see the Jiwar database section). As a result, we analyzed 40 languages using 41 distinct subtitle corpora.^1^ We cleaned the subtitle corpora in several steps to ensure data quality across languages.

First, we filtered out words not written in the target language's main script by creating a list of valid characters for each language and filtering out non-conforming words. Second, we removed stop words using language-specific lists from the open-source stopwords-iso repository.^2^ Stop words are highly frequent words that do not contribute to the meaning of a text, such as the function words “a”, “the”, “of” and “didn’t” (Sarica & Luo, 2021). Third, we implemented word length constraints to ensure that the lexical entries are individual words rather than single letters or multi-word phrases. Thus, words with less than two characters were removed (Davis, 2005). For maximum word length, we applied language-specific limits where available in the literature. For example, evidence from lexical databases suggests specific maximum word lengths for various languages: Hindi has a maximum of 17 letters, English, French, and Spanish each have 20 letters, German extends to 27 letters, and Dutch reaches 28 letters (Marian et al., 2012; Verma et al., 2022). For languages without established maximum lengths in previous studies, we adopted a conservative default limit of 25 characters, as it offered a middle ground between the maximum and minimum values observed in the literature.

Fourth, we excluded words with a frequency below 0.34 per million (Davis, 2005; Marian et al., 2012), as such words are unlikely to appear frequently and may not be part of the everyday vocabulary of most language users. Fifth, we reduced the sizes of our corpora because using a large corpus could inflate neighborhood estimates, leading to the availability of more neighbor candidates. Thus, we standardized our corpora to contain a maximum of 30,000 words in line with previous neighborhood measure calculations (e.g., Marian et al., 2012; Stein et al., 2024). Corpus sizes ranged from 4706 to 30,000 words across languages (M = 27,994.61, SD = 6355.90). Two corpora contained fewer than 10,000 words (Kazakh: 4706; Armenian: 6730), one contained fewer than 20,000 words (Afrikaans: 17,785), and the remaining corpora contained approximately 30,000 words each.

Finally, we initially considered cross-checking all corpora against established dictionaries for each respective language, using open-source, multilingual spell checkers such as Aspell,^3^ Hunspell,^4^ LanguageTool,^5^ JamSpell.^6^ However, this method proved impractical due to two reasons. First, spellcheckers may incorrectly flag real words that are not in their dictionaries (out-of-vocabulary errors), potentially leading to the unintended removal of valid but less common or inflected word forms (Mosavi Miangah, 2013; Nwesri et al., 2006). Second, several languages lacked available spellcheckers (e.g., Urdu). Given these issues, we decided to unify the cleaning process and not apply the spellchecker step across our corpora. This approach ensured consistency and allowed for comparable validation results across all languages.

#### Languages

Jiwar supports 40 distinct languages as summarized in Appendix A (Table 3). These languages represent six typological families and six writing scripts. All measures are implemented in the same way for all languages.

#### Jiwar Database: linguistic and neighborhood features

The Jiwar database contains 41 files for 40 languages. Each database file contains a column of word forms and their corresponding 24 linguistic and neighborhood characteristics. These characteristics can be grouped into three categories: linguistic information, frequency information, and neighborhood information (orthographic, phonological, and phonographic). These categories are described next.

##### Linguistic information

The database incorporates information on three linguistic measures. These include two measures of word length (numb_letters, num_phonemes) and the IPA transcription of the word. Each orthographic word in the corpora was automatically transcribed in IPA using the Python library phonemizer with eSpeak-ng^7^ as the backend software. eSpeak offers two advantages: it generates IPA for more than 100 languages and has been shown to correlate well with human-generated IPA transcriptions (Marian et al., 2012). We investigated the reliability of eSpeak IPA transcriptions using a random subset of 100 words from each of the 41 language varieties. The author manually wrote the IPA transcription for 4100 orthographic words and compared them with those generated by eSpeak. In the case the language variety is not spoken by the author (*N* = 38), the author utilized Google Translate’s text-to-speech (TTS) to identify the spoken form of the word and then transcribed it with the help of an IPA sheet. The automatic IPA transcriptions showed strong agreement with human transcriptions across languages (M Cohen’s κ = 0.89, range = 0.72–1.00), indicating excellent agreement (Gisev et al., 2013, considers κ ≥ 0.75 excellent).

##### Frequency information

The database provides information for three frequency measures: raw frequency, frequency per million words (freq_per_m), and Zipf. Raw frequency refers to the total count of the word's occurrences in the subtitle corpus. Frequency per million words was calculated using this formula: (raw frequency/total number of words in the corpus) * 1,000,000. This measure allows for comparing different-sized corpora while controlling for their size (Brezina, 2018). Zipf is a standardized frequency measure with a logarithmic scale ranging from 1 (very low-frequency words) to 7 (very high-frequency words, mostly function words). Zipf was calculated using the formula: (log10(frequency per billion words) + 3) (Van Heuven et al., 2014). The Zipf measure offers several advantages, including its ability to effectively capture the logarithmic nature of word frequencies, its ease of interpretation, and its capacity to handle words with zero frequency counts, leading to its wide adoption in most lexical databases (Soares et al., 2018; Van Heuven et al., 2014).

##### Neighborhood information

The Jiwar database provides 24 neighborhood measures calculated across three key dimensions: orthographic, phonological, and phonographic. For each dimension, six measures commonly used in the literature are included:

1. Neighborhood density (dimension_density): This field represents the number of words that differ from the target word by just one unit (letter, phoneme, or both, depending on the dimension) through substitution, addition, or deletion. Higher values indicate that the word has a higher number of similar words in the lexicon.
2. Neighbor forms (dimension_desnity_nbrs): This field provides a list of all actual neighbor words that differ from the target word by only one unit (letter, phoneme, or both, depending on the dimension) through substitution, addition, or deletion, offering concrete examples of similar words.
3. Levenshtein distance (old20; pld20; pold20): This field presents the mean of the 20 smallest Levenshtein distances to the closest words of the target word in the lexicon. The measure is referred to as OLD20 for orthographic words, PLD20 for phonological words, and POLD20 for phonographic words. Lower values indicate that the word has a higher number of similar words in the lexicon.
4. Mean neighbor frequency (dimension_nbr_zipf_m): This field includes the mean frequencies of neighboring words of the target word in each dimension.
5. SD neighbor frequency (dimension_nbr_zipf_SD): This field provides the standard deviation of the frequencies of neighboring words of the target word in each dimension.
6. Clustering coefficient (dimension_C): This field gives the degree to which the target word’s neighbors are also neighbors of one another in each dimension. The clustering coefficient C is calculated as the ratio of the actual number of links between the neighbors of a target word to all possible number of links that could exist if all neighbors were connected to each other (Batagelj & Mrvar, 1988). Values range from 0 to 1, where 0 means no neighbors are connected, and 1 indicates all neighbors are interconnected. A word with a higher clustering coefficient indicates that most of its neighbors are also neighbors of each other’s.

#### Jiwar calculator: Measures

The Jiwar calculator is an open-source, Python-based command-line interface (CLI) tool. A CLI is a text-based interface that allows users to interact with software by entering commands in a terminal (Doyle et al., 2021). CLI tools enable efficient task execution, support automation, allow future development, and perform consistently across operating systems. Given these benefits, a CLI tool was developed for the Jiwar calculator.

The Jiwar calculator uses the Jiwar database as its reference corpus, providing a built-in corpus for 41 language varieties. By utilizing a large multilingual database, the calculator allows users to analyze words and obtain detailed linguistic information quickly and efficiently for 40 languages, a feature that is currently missing from the other multilingual neighborhood calculators such as LINGUA (Westbury et al., 2007) and LexiCAL (Chee et al., 2021). The Jiwar calculator generates estimates for 49 neighborhood measures. These measures fall into three categories: orthographic, phonological, and phonographic.

##### Orthographic measures

The Jiwar calculator outputs 16 orthographic measures for each word form. The output fields return the orthographic word's number of letters (num_letters), its orthographic neighborhood size (orth_N), a list of its orthographic neighbor forms based on the N measure (orth_N_nbr), its orthographic neighborhood density value (orth_density), a list of its orthographic neighbor forms based on the density measure (orth_density_nbrs), its OLD20 value (OLD20), its orthographic clustering coefficient (orth_C), and its orthographic 2-hop density (orth_ 2hop_density). For frequency measures, it provides the mean frequency per million of orthographic neighbors (orth_nbr_fpm_m), the standard deviation of these frequencies (orth_nbr_fpm_SD), the mean frequency of higher-frequency neighbors (orth_nbr_fpm_higher_m), and the mean frequency of lower-frequency neighbors (orth_nbr_fpm_lower_m). It also includes similar measures using the Zipf scale: mean Zipf value (orth_nbr_zipf_m), standard deviation of Zipf values (orth_nbr_zipf_SD), mean Zipf of higher-frequency neighbors (orth_nbr_zipf_higher_m), and mean Zipf of lower-frequency neighbors (orth_nbr_zipf_lower_m).

Orthographic N (orth_N) counts the number of neighboring words that differ from the target word by one letter through substitution only (Coltheart et al., 1977). Orthographic neighborhood density (orth_density) count the number of neighboring words that differ from the target word by one letter through substitution, addition, or deletion (Perea, 2015). OLD20 is generated by computing the Levenshtein distance between the orthographic target word and all other orthographic words in the corpus, then taking the mean of the 20 smallest distances (Yarkoni et al., 2008). The C coefficient is calculated by determining how many of a word's orthographic neighbors are also orthographic neighbors of each other, divided by the maximum possible connections among those orthographic neighbors (Batagelj & Mrvar, 1988). The 2-hop density is calculated as the ratio of actual connections between 1-hop and 2-hop orthographic neighbors to all possible connections that could exist between them (Siew, 2017). For frequency measures, Jiwar calculates statistics about the frequencies of orthographic neighbors, defined as words differing by one letter via substitution, addition, or deletion. The calculator computes the mean and standard deviation of these neighbors' frequencies, as well as separate means for higher-frequency and lower-frequency neighbors. When using the built-in corpus, frequency calculations are provided using both frequency per million (fpm) and Zipf scales.

##### Phonological measures

The Jiwar calculator generates 17 phonological measures for each word’s IPA transcribed form. To ensure seamless computation, Jiwar incorporates an automatic IPA transcription feature for all languages supported by the eSpeak software.^8^ This functionality allows users to input word forms without their corresponding IPA transcriptions, as the system will automatically generate the required IPA transcription before computing the phonological neighborhood measures.

The resulting phonological measures include the word's IPA transcription (IPA), the number of phonemes in its IPA form (num_phonemes), its phonological neighborhood size (phon_N), a list of its phonological neighbor forms based on the N measure (phon_N_nbrs), its phonological neighborhood density value (phon_density), a list of its phonological neighbor forms based on the density measure (phon_density_nbrs), its PLD20 value (PLD20), its phonological clustering coefficient (phon_C), and its phonological 2-hop density (phon_ 2hop_density). For frequency measures, it computes the mean frequency per million of phonological neighbors (phon_nbr_fpm_m), the standard deviation of these frequencies (phon_nbr_fpm_SD), the mean frequency of higher-frequency neighbors (phon_nbr_fpm_higher_m), and the mean frequency of lower-frequency neighbors (phon_nbr_fpm_lower_m). It also calculates similar measures using the Zipf scale: mean Zipf value (phon_nbr_zipf_m), standard deviation of Zipf values (phon_nbr_zipf_SD), mean Zipf of higher-frequency neighbors (phon_nbr_zipf_higher_m), and mean Zipf of lower-frequency neighbors (phon_nbr_zipf_lower_m). These phonological measures are defined similarly to their orthographic counterparts, with the key difference being the use of the phoneme as the unit of analysis input rather than the letter.

##### Phonographic measures

The Jiwar calculator produces 16 phonographic measures for each word form. Since these measures require the IPA form of the target word, Jiwar automatically transcribes all target words into the necessary IPA transcription for languages supported by the eSpeak software. Jiwar gives the word form’s IPA transcription (IPA), the word's phonographic neighborhood size (pg_N), a list of its phonographic neighbor forms based on the N measure (pg_N_nbrs), its phonographic neighborhood density value (pg_density), a list of its phonographic neighbor forms based on the density measure (pg_density_nbrs), its PGLD20 value (PGLD20), its phonographic clustering coefficient (pg_C), and its phonographic 2-hop density (pg_ 2hop_density). For frequency measures, it determines the mean frequency per million of phonographic neighbors (pg_nbr_fpm_m), the standard deviation of these frequencies (pg_nbr_fpm_SD), the mean frequency of higher-frequency neighbors (pg_nbr_fpm_higher_m), and the mean frequency of lower-frequency neighbors (pg_nbr_fpm_lower_m). It also derives similar measures using the Zipf scale: mean Zipf value (pg_nbr_zipf_m), standard deviation of Zipf values (pg_nbr_zipf_SD), mean Zipf of higher-frequency neighbors (pg_nbr_zipf_higher_m), and mean Zipf of lower-frequency neighbors (pg_nbr_zipf_lower_m). These phonographic measures are defined similarly to their orthographic counterparts, with the exception of using both letters and phonemes as the units of analysis rather than letters alone.

#### How to use Jiwar

##### The Jiwar database

Users can access and download the Jiwar database from its main GitHub page in any of the following file formats (.tsv,.csv,.xlsx, and.txt).^9^ Users can download all databases or a language-specific database. We recommend users to download and use the database in Excel format as this format offers many filtering options and allows ease of data retrieval. For example, if the user wants to find all words with a specific number of letters, the user can easily do so in three steps. First, open the target Excel file and locate the column labeled “number_of_letters”. Second, click on the filter button at the top of this column. Third, in the filter menu, select the specific number of letters the user is interested in, such as 5. Excel will then display only the five-letter words in the database. The user can further refine her search by applying additional filters to other columns, such as frequency or phonological measures.

##### The Jiwar calculator

**Fig. 1:**
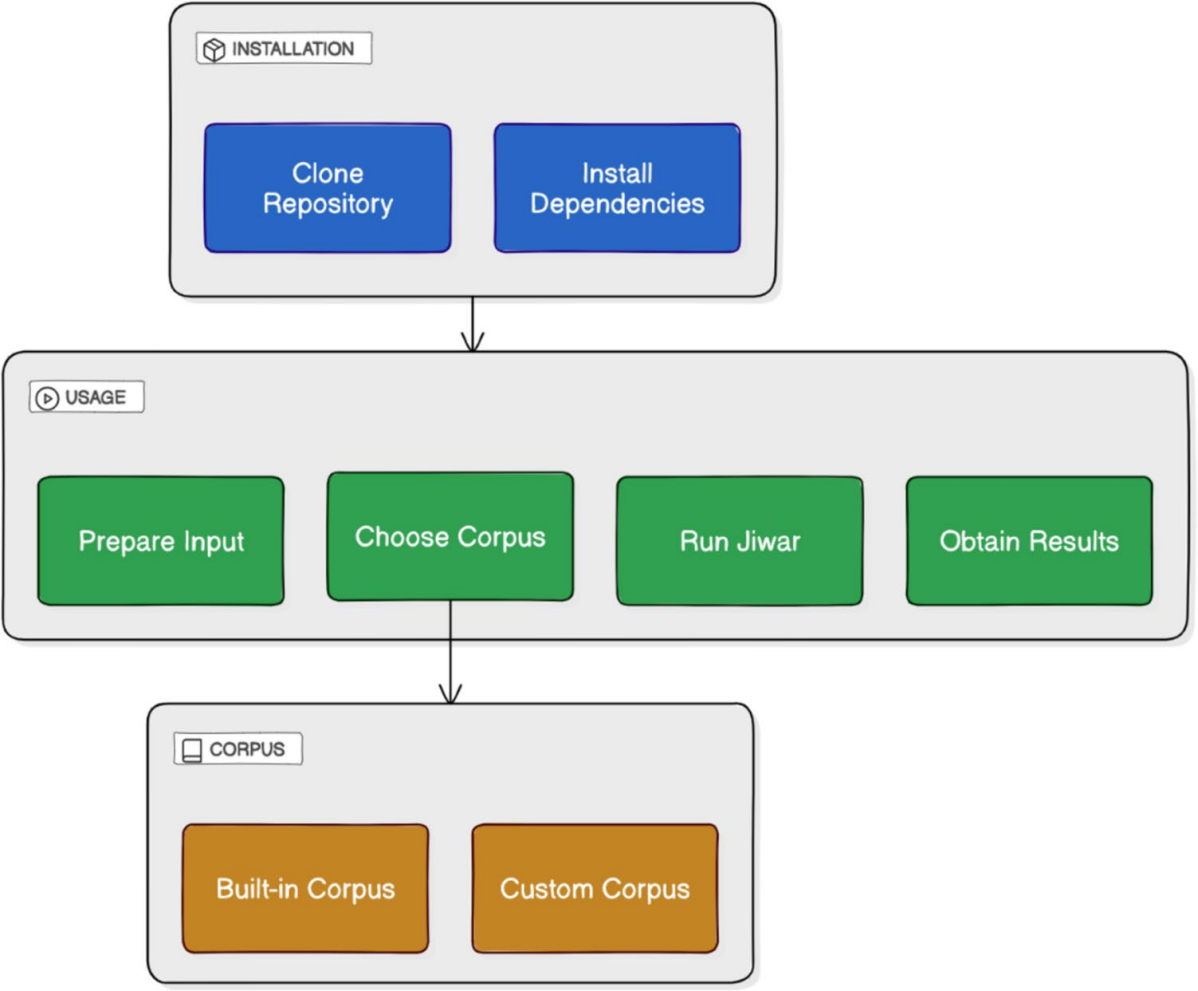
Jiwar calculator installation and usage workflow

The calculator can be used to generate metrics for words and nonwords. There are two main ways to use the Jiwar calculator: online without installation or via a local installation on the user’s device. For the no-installation option, users can use the interactive Google Colab notebook available on Jiwar's GitHub repository, which includes a step-by-step description of how to run the calculator. For the local installation option (Fig. 1), users are recommended to follow the next steps:

1. Prepare the input file (csv, xlsx, tsv, or txt) with at least a 'word' column.
2. For large files (> 10,000 words), split into smaller files for faster processing.
3. Use the accepted writing form for each language (see Jiwar's GitHub for a list of languages and their supported writing form^10^).
4. Note that the calculator is case-insensitive but recognizes diacritics.
5. Place the input file in the '/data/input' folder.
6. Run the tool: python jiwar.py
7. Follow prompts to select language, input file, and desired measures.
8. Choose corpus option:
9. Built-in corpus (recommended for supported languages)
10. Custom corpus (for unsupported languages or specific needs)
  Prepare a.csv or.xlsx file with a “word” column
  Optional: Add frequency and IPA columns (“frequency_1”, “frequency_2”, “IPA”)
  Place the custom corpus in “data/corpus/user_loaded/” directory
11. Jiwar will process the input and save results to a csv file in '/data/output'.

To enhance user experience and ensure software stability, the Jiwar CLI tool incorporates detailed help messages and robust error handling. The tool provides various help messages to guide users through its features. The tool also outputs clear warning messages when issues arise during execution, such as incorrect file formats, misnamed columns, or unsupported languages (see Appendix B (Table 4)). This comprehensive approach to error management helps users quickly identify and resolve problems, ensuring better tool performance. An online documentation website is also available for the Jiwar calculator,^11^ providing more details and examples.

#### Licensing

Jiwar is an open-source software tool released under the GNU General Public License v3.0. This license grants researchers the freedom to use, modify, and share the software, encouraging collaboration and knowledge sharing within the psycholinguistic community. The dependency of Jiwar is phonemizer, and it is licensed under the GNU General Public License v3.0.

### Validation of the Jiwar calculator

This section examines the validity of the Jiwar calculator. A common method for assessing the validity of automatic tools involves investigating their intrinsic validity (Lu, 2010; McNamara et al., 2014). Intrinsic validity refers to the inherent ability of a tool to measure what it purports to measure. In linguistic research, the intrinsic validity of automatic tools can be evaluated using two approaches: instrument reliability and instrument validity (Linders & Louwerse, 2023).

Instrument reliability assesses the degree to which measurements obtained from the tool correlate well with those calculated by human experts or gold-standard databases (e.g., Lu, 2010). Gold-standard datasets are the best available benchmarks, against which new tools are compared against (Cardoso et al., 2014; Versi, 1992). In this analysis, a significant positive correlation indicates that the tool consistently produces results that match established benchmarks, thereby confirming its reliability. Very few neighborhood calculators have been assessed for instrument reliability (see Table 2). For instance, LexiCAL was validated by comparing its measures against the neighborhood measures reported in two large English datasets: the Hoosier Mental Lexicon (Nusbaum et al., 1984) and the English Lexicon Project (Balota et al., 2007). Results indicated that most measures correlated positively and significantly, with coefficients ranging from 0.97 to 1.00 (Chee et al., 2021).

On the other hand, instrument validity examines the tool's ability to accurately capture and predict the target linguistic effect it was designed to measure (e.g., Hwang & Kim, 2023). This aspect of validation ensures that the tool fulfills its intended purpose. A significant predictive ability suggests strong instrument validity. Most neighborhood calculators’ instrument validity has not been sufficiently examined (see Table 2). An exception is Storkel and Hoover’s (2010). In this study, the authors validated a calculator of English phonological neighborhood density (PND) as a measure for differentiating neighborhood effects across two speaker groups. The authors compared the calculator’s PND values based on child corpora versus adult corpora. Findings revealed a significant difference: child PND values were consistently lower than adult PND values, particularly for words in denser neighborhoods. This result is consistent with theoretical evidence, demonstrating the calculator's ability to accurately capture the target linguistic effect and establishing its instrument validity.

In this study, we validated the Jiwar calculator through a two-step process: first, by assessing its instrument reliability, and second, by evaluating its instrument validity. Both steps involved utilizing the Lexicon Project databases and other comparable large-scale linguistic datasets. Lexicon projects are comprehensive studies that collect behavioral and linguistic data for a large number of words in a target language through experiments such as lexical decision and naming tasks (Keuleers & Balota, 2015). We used all available databases that allowed us to correlate or predict comparable measures across the existing datasets and Jiwar’s measures. These included datasets for American English (Balota et al., 2007), British English (Keuleers et al., 2012), German (Busch et al., 2021), Spanish (Aguasvivas et al., 2018; Duchon et al., 2013), Brazilian Portuguese (Estivalet & Meunier, 2015), Catalan (Boada et al., 2020; Guasch et al., 2023), French (Ferrand et al., 2010, 2011; New, 2006), Dutch (Brysbaert et al., 2019; Speed & Brysbaert, 2024), and Italian (Goslin et al., 2014).

#### Instrument reliability: Correlations with prior databases

Two neighborhood measures are widely reported in existing datasets: OLD20 and, to a lesser extent, PLD20. As such, for each of these two measures, we performed correlations to compare values from prior studies with corresponding values generated by the Jiwar calculator. See Appendix C (Table 5) for correlation statistics.^12^

**Fig. 2:**
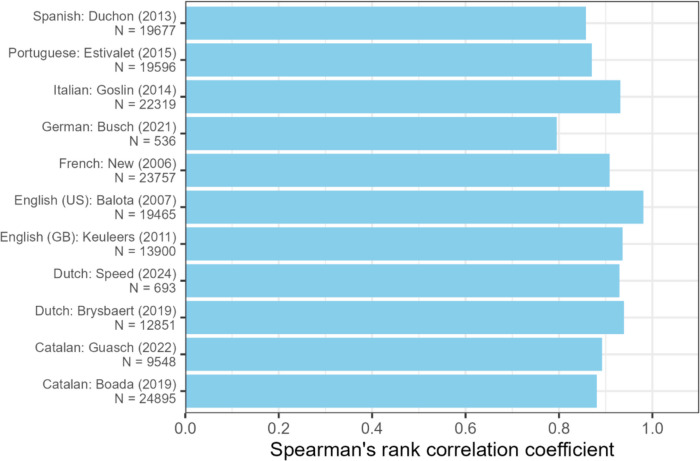
Spearman's rank correlation between OLD20 values from prior studies and OLD20 values generated by the Jiwar calculator for a common set of words

Figure 2 shows the adjusted correlation between Jiwar’s OLD20 values and the values reported by previous databases. Correlations with 11 language datasets (M = 16,285 words, range: 536–24,895 words) yielded Spearman coefficients ranging from 0.79 to 0.98, suggesting strong correlations (Schober et al., 2018). Crucially, OLD20 values from small (German: Busch et al., 2021; Dutch: Speed & Brysbaert, 2024) and large datasets (Catalan: Boada et al., 2020; Italian: Goslin et al., 2014) equally showed positive correlations with Jiwar’s OLD20 values, indicating the reliability of the Jiwar calculator across distinct languages and dataset sizes.

**Fig. 3:**
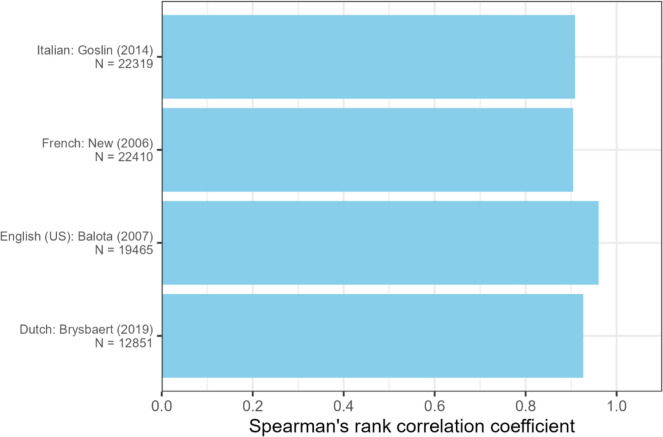
Spearman's rank correlation between PLD20 values from prior studies and PLD20 values generated by the Jiwar calculator for a common set of words

Figure 3 shows the adjusted correlation between Jiwar’s PLD20 values and the values reported by prior databases. Similar to OLD20 results, correlations for the PLD20 measure were positive and statistically significant across all databases, including Dutch (ρ = 0.93), English (ρ = 0.96), French (ρ = 0.90), and Italian data (ρ = 0.91).

#### Instrument validity: Predicting behavioral data

We conducted a series of multiple linear regression models to evaluate the predictive power of several neighborhood measures known to impact behavioral results in two gold-standard visual word recognition tasks: the visual lexical decision task and the visual naming task (Coltheart et al., 2001; Dujardin & Mathey, 2020; Ferrand et al., 2011; Grainger & Jacobs, 1996; Perea & Rosa Martínez, 2000). Gold-standard tasks refer to tasks that have been thoroughly examined and have a reputation in the field as a reliable experimental method (Cardoso et al., 2014). The examined neighborhood measures are OND, OLD20, PLD20, and orthographic C. Using such visual word recognition tasks, prior research has shown a positive effect of OND (e.g., Duñabeitia & Vidal-Abarca, 2008; V. J. Laxon et al., 1988; for a review, Perea & Rosa Martínez, 2000), OLD20 (e.g., Yarkoni et al., 2008), PLD20 (e.g., Yap & Balota, 2009), and orthographic C on the speed and/or accuracy of word recognition (e.g., Siew, 2018). It should be noted that although these neighborhood measures were generally significant for English behavioral data, they sometimes emerged as non-significant predictors in other languages (e.g., Ferrand et al., 2011; Maziyah Mohamed et al., 2023). For instance, in French, OLD20 showed a non-significant effect in the visual lexical decision task and the visual naming task. Likewise, in Malay, OLD20 may exhibit a non-significant influence in the visual lexical decision task, although this effect may be triggered by the morphological structure of the selected experimental words.

All models were constructed in two steps. Initially, a baseline model was built using two established predictors in lexical processing: word frequency (e.g., Brysbaert & New, 2009; Ferrand et al., 2011; Forster & Chambers, 1973; Hudson & Bergman, 1985) and word length (e.g., Keuleers et al., 2012; New et al., 2007). Subsequently, each target neighborhood measure was introduced into the baseline model in a separate analysis. This approach enabled us to evaluate each measure's unique contribution to explaining variance in visual word recognition (e.g., Siew, 2018; Siew & Vitevitch, 2019; Yap & Balota, 2009). Visual lexical decision and visual naming data were retrieved from six lexicon projects for words that appeared in both the Jiwar database and these projects (see the Validation section). All target neighborhood measures were derived from Jiwar and added as predictors in the models. We used adjusted *R*^2^ for all model comparisons, as this metric accounts for the number of predictors, providing a robust measure for model comparison (Levshina, 2015).

##### Visual lexical decision task

**Fig. 4:**
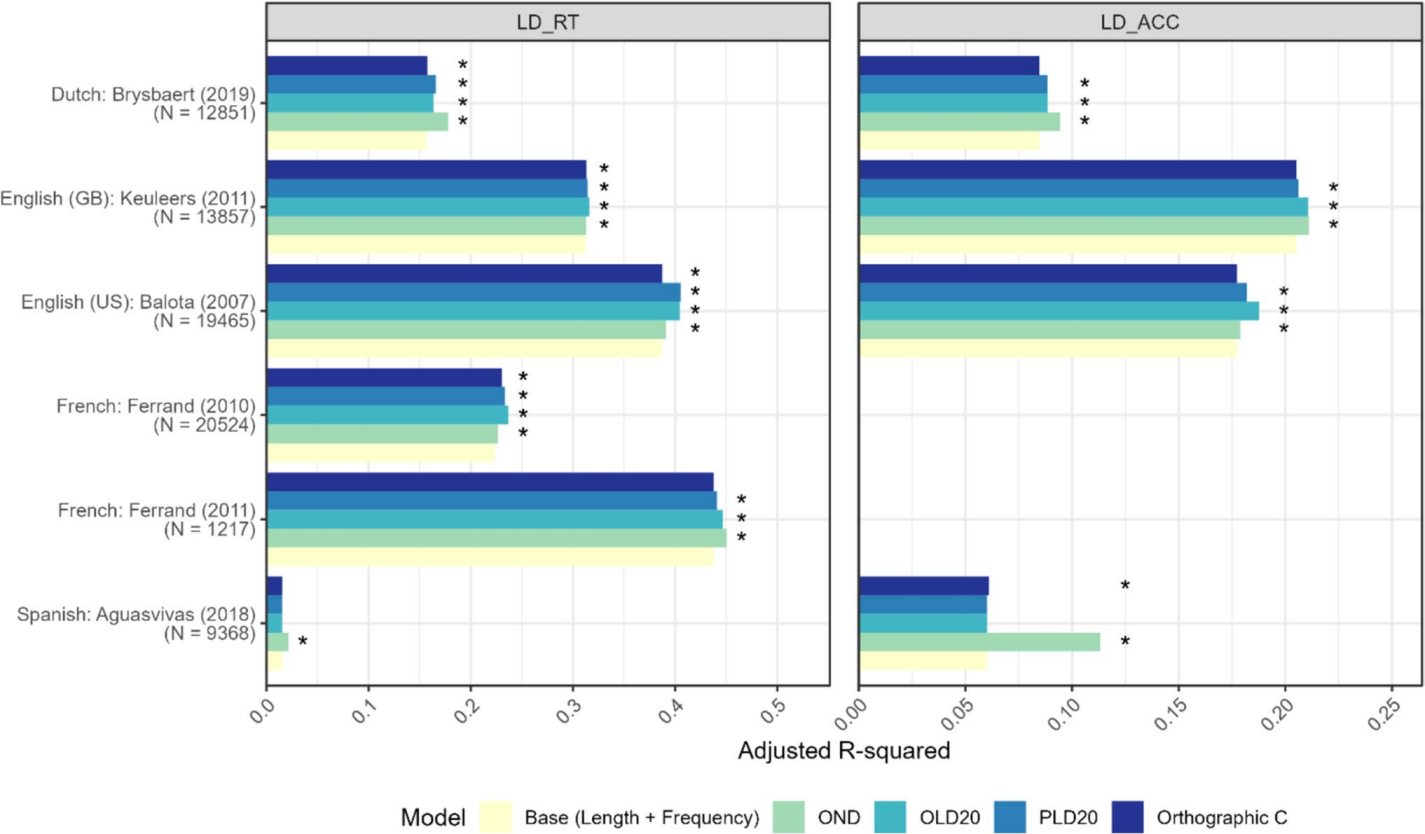
Variance explained for LD data. *OND*, orthographic neighborhood density; *OLD20*, orthographic Levenshtein distance 20; *PLD*, phonological Levenshtein distance 20; *Orthographic C*, orthographic clustering coefficient C

Figure 4 illustrates the variance explained (*R*^2^) in visual lexical decision (LD) response times and accuracy by four selected Jiwar neighborhood measures. See Appendix D (Table 6) for detailed statistics. For LD reaction time data, all Jiwar neighborhood measures consistently outperformed the baseline model in explaining variance for most datasets, including Dutch, English (British and American variants), and French. In contrast, only OND surpassed the baseline model in explaining variance for the Spanish dataset. The Spanish words may have similar OLD20/PLD20 values, possibly reducing the measures’ variability (e.g., Ferrand et al., 2011). Nevertheless, it is difficult to interpret the Spanish results since the publication of the Spanish dataset did not involve comprehensive analyses of LD and NMG data (Aguasvivas et al., 2018), limiting our understating of how neighborhood measures may influence behavioral results.

For LD accuracy data, the Jiwar measures OND, OLD20, and PLD20 significantly accounted for more explained variance than the base model in Dutch and English (British, American) but not for Spanish data. Notably, the orthographic C measure contributed less uniquely to LD accuracy data compared to its impact on LD reaction time data across the examined language varieties (e.g., Siew, 2018).

##### Visual naming task

**Fig. 5:**
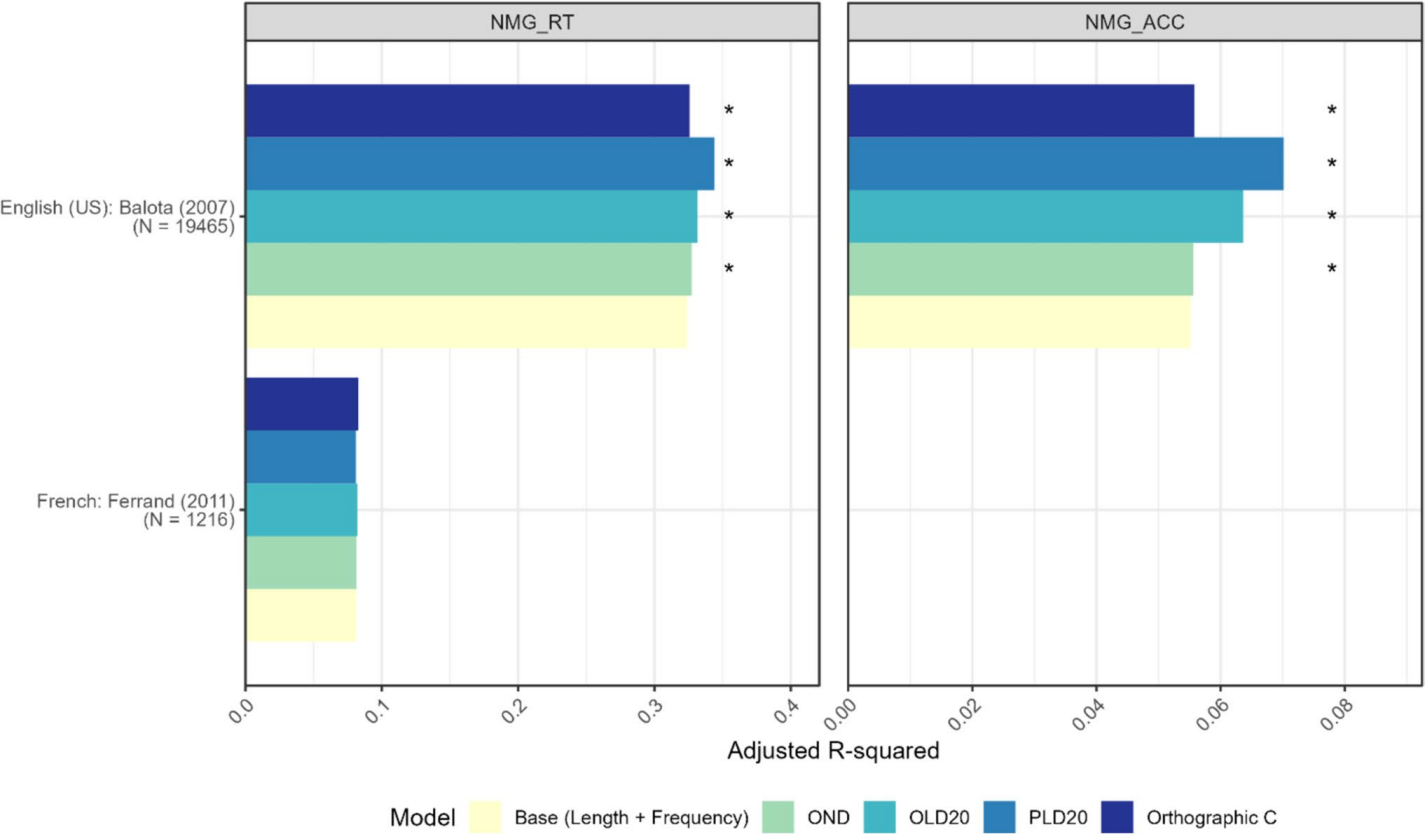
Variance explained for NMG data. *OND*, orthographic neighborhood density; *OLD20, *orthographic Levenshtein distance 20; *PLD*, phonological Levenshtein distance 20; *Orthographic C*, orthographic clustering coefficient C

Figure 5 illustrates the variance explained (*R*^2^) in visual naming (NMG) response times and accuracy by four selected Jiwar neighborhood measures. See Appendix E (Table 7) for detailed statistics. The results are derived from multiple linear regression models with quadratic terms for the predictor to account for the potential non-linear neighborhood effects on NMG data (Hendrix et al., 2019). In the English dataset, all neighborhood measures explained a greater proportion of variance in NMG reaction times and accuracy compared to the baseline model. However, in the French dataset, none of these measures outperformed the baseline. The discrepancy in NMG results between French and English may arise from factors such as limited variability in the measures’ values and/or smaller word entries in the French dataset (Ferrand et al., 2011). However, these explanations alone cannot fully account for the substantial findings in French LD data for the same dataset (Ferrand et al., 2011), suggesting a combination of task-specific (LD vs. NMG) and dataset-related influences.

To summarize, the validation study demonstrated the instrument reliability and instrument validity of the Jiwar calculator. We found significant positive correlations between Jiwar's OLD20/PLD20 values and those from existing databases representing four to 11 languages, indicating excellent reliability. Three Jiwar measures (OND, OLD20, PLD20) consistently outperformed baseline models in explaining variance for LD and NMG tasks across several language datasets. These findings are consistent with results from prior experimental research. The orthographic C measure showed variable results, with its contribution fluctuating based on language, task type (LD vs. NMG), and target-dependent variable (RT vs. ACC). Overall, these results suggest that the Jiwar calculator is a reliable and valid tool for generating neighborhood measures in a wide range of languages.

## Discussion

This study introduced an open-source word neighborhood database and an accompanying calculator, currently supporting 40 languages. The Jiwar database includes information for 24 neighborhood measures, encompassing linguistic, frequency, and neighborhood information. The Jiwar calculator offers users the choice to generate any of its 46 neighborhood measures, categorized into three categories: orthographic, phonological, and phonographic. The calculator can be used to generate measures for words and nonwords in the supported languages. The calculator is implemented as an open-source Python CLI tool, with an aim to offer flexibility for future updates including the addition of further languages and neighborhood measures. Compared to previous calculators, the Jiwar calculator provides two key advantages: a built-in multilingual database for 40 languages and a function to generate IPA transcription for more than 100 language varieties. The Jiwar database is embedded in the calculator, allowing users to rapidly obtain 24 linguistic and neighborhood measures for the target language without the need to prepare a specific database. Further, the calculator provides the automatic generation of IPA transcription for currently supported language and for 90 additional languages that do not have a built-in corpus, facilitating research on phonological neighborhood effects across various languages.

We conducted a two-step validation study to assess the effectiveness of the Jiwar calculator. First, we correlated the values of two common neighborhood measures (OLD20, PLD20) based on Jiwar calculations and various large-scale datasets across several languages. Results showed positive significant correlations with prior datasets, indicating the great reliability of the introduced calculator. Second, we used four widely utilized neighborhood measures generated by the calculator (OND, OLD20, PLD20, and orthographic C) to predict behavioral results from two gold-standard visual word recognition tasks: LD and NMG tasks. Based on multiple-linear regression analysis of several language datasets, we found that three of the examined Jiwar measures (OND, OLD20, PLD20) consistently showed unique variance for LD and NMG tasks across a number of languages. Combined, these findings suggest the reliability and validity of the Jiwar calculator, indicating that it can serve as a valuable multilingual resource for the generation of neighborhood measures for words and nonwords across a large range of languages.

Nevertheless, the Jiwar calculator was validated based on a subset of the supported languages. This was largely due to the lack of large-scale linguistic and psycholinguistic datasets for less-explored languages, and sometimes due to the limited availability of neighborhood information in existing datasets. However, we have taken significant steps to minimize the potential for errors in the languages not included in the validation process. We followed the same corpus pre-processing steps for all languages and developed the same scripts for calculating the neighborhood measures across all languages, reducing the likelihood of language-specific errors. Further, it is important to note that our validation efforts were constrained to examining only four out of the 46 measures provided by the Jiwar calculator. While most Jiwar calculator’s measures are commonly used in experimental research either as predictors or controlling variables, not all datasets report metrics for these measures. Nevertheless, Jiwar's neighborhood measures are interconnected and built upon one another, suggesting a high likelihood that all measures will provide consistent results aligning with those examined for validity. For instance, the measure N (short for Coltheart’s N) considers only neighbors differing by one unit (letter/phoneme) via substitution, while the measure neighborhood density expands this definition to include neighbors differing by one unit through substitution, addition, or deletion. We hope that the introduction of Jiwar will allow researchers to generate neighborhood information and examine their potential effects in a wider number of languages, and these findings can in turn support the design and validation of future multilingual calculators.

The Jiwar database is available as standalone Excel files to facilitate information query. These files are free to download from the official Jiwar GitHub repository. The Jiwar calculator is a Python CLI tool that can be used via a local installation the user’s machine using any standard code editor (e.g., Visual Studio Code, PyCharm, Sublime Text, Atom) or via a web-based Jupyter notebook. Detailed instructions for downloading, installing, and using both the database and calculator are provided in Jiwar’s online documentation.^13^

On the other hand, a reviewer suggested that the introduced calculator can also be used to compute measures for cross-linguistic phonological neighbors. In other words, the calculator may be utilized to examine the number of Spanish words which sound like a given English word. For example, the English word “key” and the Spanish word “sí” are phonological neighbors since they differ by the substitution of the single phoneme /s/ for /k/. Research suggests that between 2 and 12% of words in some Indo-European languages (e.g., French) have at least one phonological neighbor in another typologically-related language (e.g., English) (Marian et al., 2012; Vitevitch, 2012). One existing cross-linguistic neighborhood calculator in the literature is CLEARPOND (Marian et al., 2012). In this study, Marian et al. examined cross-linguistic phonological neighbors in five common Indo-European languages: Dutch, English, French, German, and Spanish. They reported that around 11% of Dutch words, 12% of French words, 12% of German words and 1.6% of Spanish words had one or more English words as phonological neighbors. Similarly, Vitevitch (2012) analyzed two Spanish corpora and found that 2% to 2.5% of Spanish words had one or more English words as phonological neighbors. However, the Jiwar calculator was not designed to measure cross-linguistic phonological neighbors. Unlike CLEARPOND, which removed foreign words from each language corpus using dictionaries, we did not clean our corpora using dictionaries for reasons stated in the Methods section. As such, generating cross-linguistic phonological neighborhood measures via the Jiwar calculator could yield inflated values especially for languages written in the same alphabet (e.g., English and Spanish). Therefore, we do not recommend using the Jiwar calculator to compute cross-linguistic phonological neighborhood measures.

Finally, the regression analysis revealed some variability in neighborhood effects across tasks. We examined two tasks: a visual lexical decision task (LD) and a word naming task (NMG). The analysis focused on datasets from two languages: English and French. In the English dataset (Balota et al., 2007), the examined neighborhood measures explained significant variance in both LD and NMG reaction times and accuracy. However, in the French dataset (Ferrand et al., 2011), these measures only accounted for variance in LD reaction times, showing no significant effect on NMG. This variability may be attributed to task effects. Prior studies have observed a difference in the neighborhood effect across tasks (e.g., Balota et al., 2004; Stein et al., 2024; Yap et al., 2010). In English, Balota et al. (2004) have found that neighborhood size had a stronger influence on NMG reaction times than on LD reaction times. In Malay, both neighborhood size and OLD20 were reported to influence LD response times but did not exhibit a significant effect on NMG response times (Yap et al., 2010). In Hebrew, OLD20 were found to be a significant predictor of LD response times and accuracy, but it only influenced accuracy in NMG (Stein et al., 2024). These findings suggest that LD and NMG tasks may engage distinct cognitive processing mechanisms. A thorough discussion of the task effect is out of the scope of the paper, and further research is needed to examine this effect in several languages to provide robust evidence.

Although this study designed and validated a multilingual linguistic tool, this study is limited in several aspects. First, even though Jiwar supports 40 languages utilizing six distinct writing systems (Latin, Arabic, Cyrillic, Greek, Armenian, Hangul), it only covers a small number of the world’s languages. However, the Python scripts for the Jiwar CLI tool are open-source and can be readily adapted to generate neighborhood measures for other languages. Second, corpus sizes varied across languages. While most languages had approximately 30,000 words, two languages (Kazakh and Armenian) had considerably smaller corpora, with fewer than 10,000 words each. Smaller corpus sizes could potentially distort neighborhood estimates. Future research should aim to use larger corpora for under-resourced languages and validate the Jiwar calculator’s performance across both small and large databases. Third, the built-in corpus is based on movie/TV subtitles and may not be suitable for examining neighborhood effects in populations such as children, individuals with disorders, and second language learners. The incorporation of diverse corpora options could expand the tool's usefulness across various speaker groups.

An additional limitation is that while the current measures are comprehensive, they do not account for transposition neighbors. Letter-transposed neighbors are word pairs that share the same letters but have two swapped letters, such as silver-sliver, from-form, and trial-trail (Johnson et al., 2024). The classical Levenshtein distance counts these transpositions as two separate substitution steps, whereas the Damerau-Levenshtein distance treats them as a single transposition step (Hosangadi, 2012). Empirical evidence suggests that transposed-letter neighbors are activated during visual word recognition and reading, indicating that they constitute one aspect of a word’s neighborhood (Perea, 2015). This suggests the need to consider the effect of transposed neighbors in subsequent studies.

## Conclusion

The present study introduced and validated a multilingual database and calculator for neighborhood measures across three dimensions (orthographic, phonological, and phonographic) covering 40 languages. The introduction of this resource could facilitate research on understudied languages, addressing the long-standing issue of overreliance on WEIRD samples in linguistic and psycholinguistic research. The Jiwar database and calculator are both open-source, allowing researchers to expand and modify the calculator’s functionality to suit their needs.

## Data Availability

All Jiwar databases are available at GitHub: https://github.com/AlaaAlzahrani/Jiwar_database. All datasets used for the analysis are available at OSF: https://osf.io/psrav/?view_only=1cde0e02e3aa47189fa5764aac3916da.

## Appendix 1

**Table 3:** Jiwar's supported languages and their corresponding scripts

N	Code	Language	Supported Writing Script	Language Family
1	af	Afrikaans	Latin	Indo-European
2	ar	Arabic	Fully diacritized Arabic	Semitic
3	bg	Bulgarian	Cyrillic	Indo-European
4	bs	Bosnian	Cyrillic, Latin	Indo-European
5	ca	Catalan	Latin	Indo-European
6	cs	Czech	Latin	Indo-European
7	de	German	Latin	Indo-European
8	el	Greek	Greek	Indo-European
9	en-gb	English (GB)	Latin	Indo-European
10	en-us	English (US)	Latin	Indo-European
11	eo	Esperanto	Latin	NA*
12	es	Spanish	Latin	Indo-European
13	et	Estonian	Latin	Uralic
14	eu	Basque	Latin	Language Isolate*
15	fa	Persian	Perso-Arabic	Indo-European
16	fi	Finnish	Latin	Uralic
17	fr	French	Latin	Indo-European
18	hr	Croatian	Latin	Indo-European
19	hu	Hungarian	Latin	Uralic
20	hy	Armenian	Armenian	Indo-European
21	id	Indonesian	Latin	Austronesian
22	it	Italian	Latin	Indo-European
23	kk	Kazakh	Cyrillic	Turkic
24	ko	Korean	Hangul	Koreanic
25	lt	Lithuanian	Latin	Indo-European
26	lv	Latvian	Latin	Indo-European
27	mk	Macedonian	Cyrillic	Indo-European
28	ms	Malay	Latin	Austronesian
29	nl	Dutch	Latin	Indo-European
30	no	Norwegian	Latin	Indo-European
31	pl	Polish	Latin	Indo-European
32	pt	Portuguese	Latin	Indo-European
33	ro	Romanian	Latin	Indo-European
34	ru	Russian	Cyrillic	Indo-European
35	sk	Slovak	Latin	Indo-European
36	sq	Albanian	Latin	Indo-European
37	sr	Serbian	Cyrillic	Indo-European
38	sv	Swedish	Latin	Indo-European
39	tr	Turkish	Latin	Turkic
40	uk	Ukrainian	Cyrillic	Indo-European
41	ur	Urdu	Perso-Arabic	Indo-European

NA = not applicable; Language Isolate = a term that refers to languages that have not yet shown a genetic relationship with any other languages

## Appendix 2

**Table 4:** Error messages in Jiwar's calculator: Meaning and troubleshooting solutions

Error message	Meaning	Solution
Unsupported language: {language_input}	The specified language is not supported by the tool	Check the list of supported languages and use a valid language code or name. You can view the supported languages by running the get_supported_languages_info() function
User corpus file not found: {user_corpus_file}	The user-specified corpus file could not be found in the expected directory	Ensure the corpus file is in the correct directory (user_loaded folder) or provide the full path to the file. Double-check the filename and path for typos
User corpus file not found: {user_corpus_file}	The built-in subtitle corpus for the specified language is missing	Use a custom corpus instead, or check if there is an updated version of Jiwar with the missing corpus file
Unable to determine the correct encoding for file {file_path}	The tool could not automatically detect the file encoding	Try opening the file in a text editor and saving it with a standard encoding like UTF-8. Then retry loading the file
Error reading Excel file {file_path}: {str(e)}	There was an issue reading the Excel file provided as corpus	Ensure the Excel file is not corrupted and is in a compatible format (.xlsx). Try saving it as a CSV file instead and use that
The custom corpus is missing the required 'word' column	The user's custom corpus does not have a column named 'word'	Add a column named 'word' to your custom corpus file containing the words in your language
The corpus is missing the frequency column: {freq_col}	A specified frequency column is not present in the corpus	Ensure your corpus includes the necessary frequency columns, or update your code to use the correct column names
The '{freq_col}' column contains non-numeric values	The frequency column in the corpus contains non-numeric data	Check your corpus file and ensure all values in the frequency columns are numeric. Remove or correct any non-numeric entries
No corpus loaded or corpus is empty	Attempted to use corpus data before loading it or the loaded corpus is empty	Make sure to load a valid, non-empty corpus before performing operations that require it
Frequency columns not set. Ensure corpus is loaded and validated	Tried to access frequency columns before they were set	Load and validate the corpus before attempting to access frequency columns
File not found: {filename}	The input file specified by the user was not found	Check that the file exists in the specified location. If using just a filename, ensure it is in the current directory or Jiwar's input directory
The input file contains no data	The input file was found but is empty	Check your input file and ensure it contains the necessary data
The input file must contain a 'word' column	The input file is missing a required 'word' column	Add a 'word' column to your input file containing the words you want to analyze
All rows were removed during cleaning. Please check your input file	After cleaning, no valid data remained in the input file	Review your input file. Ensure it contains valid data and that words are not empty or null
It looks like eSpeak is not installed on your system	The eSpeak tool required for IPA generation is not installed	Follow the instructions provided in the error message to install eSpeak on your system
Unsupported language. Please try again	The language entered is not recognized by Jiwar	Review the list of supported languages provided and enter a valid language code or name
Error loading corpus: {e}	There was a problem loading the specified corpus	Check the corpus file for issues. Ensure it is in the correct format and location. Review any specific error details provided
Error: {e} (when reading input file)	The input file could not be read successfully	Verify the file exists in the specified location. Check file permissions and format. Ensure the file is not corrupted
Error reading input file: {e}	There was a problem processing the contents of the input file	Review the file contents. Ensure it is in the expected format and contains valid data
Invalid input. Please enter 'all', 'orth', 'phon', 'pg', or a combination of these	The measure types entered are not recognized	Enter only valid measure types as specified in the prompt

## Appendix 3

**Table 5:** Correlations with prior databases for two-word neighborhood measures

Dataset	Measure	Correlation method	Coefficient	95% CI	*p*	Obs
Catalan: Boada (2019)	OLD20	Pearson	0.881734913	[0.88, 0.88]	0	24,895
Catalan: Guasch (2022)	OLD20	Pearson	0.881917567	[0.88, 0.89]	0	9548
Dutch: Brysbaert (2019)	OLD20	Pearson	0.932656544	[0.93, 0.93]	0	12,851
Dutch: Brysbaert (2019)	PLD20	Pearson	0.915919063	[0.91, 0.92]	0	12,851
Dutch: Speed (2024)	OLD20	Pearson	0.92681298	[0.92, 0.94]	3.88E-296	693
English (GB): Keuleers (2011)	OLD20	Pearson	0.945468767	[0.94, 0.95]	0	13,900
English (US): Balota (2007)	OLD20	Pearson	0.979582618	[0.98, 0.98]	0	19,465
English (US): Balota (2007)	PLD20	Pearson	0.950438863	[0.95, 0.95]	0	19,465
French: New (2006)	OLD20	Pearson	0.91498524	[0.91, 0.92]	0	23,757
French: New (2006)	PLD20	Pearson	0.913713546	[0.91, 0.92]	0	22,410
German: Busch (2021)	OLD20	Pearson	0.770178129	[0.73, 0.80]	2.31E-106	536
Italian: Goslin (2014)	OLD20	Pearson	0.933718878	[0.93, 0.94]	0	22,319
Italian: Goslin (2014)	PLD20	Pearson	0.912274139	[0.91, 0.91]	0	22,319
Portuguese: Estivalet (2015)	OLD20	Pearson	0.869404198	[0.87, 0.87]	0	19,596
Spanish: Duchon (2013)	OLD20	Pearson	0.869523395	[0.87, 0.87]	0	19,677
Catalan: Boada (2019)	OLD20	Spearman	0.880907299	[0.88, 0.88]	0	24,895
Catalan: Guasch (2022)	OLD20	Spearman	0.892328736	[0.89, 0.90]	0	9548
Dutch: Brysbaert (2019)	OLD20	Spearman	0.939015529	[0.94, 0.94]	0	12,851
Dutch: Brysbaert (2019)	PLD20	Spearman	0.926677231	[0.92, 0.93]	0	12,851
Dutch: Speed (2024)	OLD20	Spearman	0.929433026	[0.92, 0.94]	2.10E-301	693
English (GB): Keuleers (2011)	OLD20	Spearman	0.93614335	[0.93, 0.94]	0	13,900
English (US): Balota (2007)	OLD20	Spearman	0.980702451	[0.98, 0.98]	0	19,465
English (US): Balota (2007)	PLD20	Spearman	0.960467781	[0.96, 0.96]	0	19,465
French: New (2006)	OLD20	Spearman	0.908489145	[0.91, 0.91]	0	23,757
French: New (2006)	PLD20	Spearman	0.904309654	[0.90, 0.91]	0	22,410
German: Busch (2021)	OLD20	Spearman	0.795176325	[0.76, 0.82]	4.19E-118	536
Italian: Goslin (2014)	OLD20	Spearman	0.931509519	[0.93, 0.93]	0	22,319
Italian: Goslin (2014)	PLD20	Spearman	0.908691755	[0.91, 0.91]	0	22,319
Portuguese: Estivalet (2015)	OLD20	Spearman	0.870469132	[0.87, 0.87]	0	19,596
Spanish: Duchon (2013)	OLD20	Spearman	0.857831666	[0.85, 0.86]	0	19,677

Obs. = number of matched words across the Jiwar database and the corresponding dataset

## Appendix 4

**Table 6:** Multiple regression model results for lexical decision data

Dataset	Model	*R* ^2^	Adj. R^2^	AIC	BIC	Obs	*F*	*p*	Sig	DV
English (GB): Keuleers (2011)	Base (Length + Frequency)	0.312672	0.312573	150,159.4	150,189.6	13,857				LD_RT
English (GB): Keuleers (2011)	OND	0.313084	0.312935	150,153.1	150,190.8	13,857	8.311878	0.003945	TRUE	LD_RT
English (GB): Keuleers (2011)	OLD20	0.316194	0.316046	150,090.2	150,127.9	13,857	71.35266	3.28E-17	TRUE	LD_RT
English (GB): Keuleers (2011)	PLD20	0.314355	0.314206	150,127.4	150,165.1	13,857	34.00266	5.63E-09	TRUE	LD_RT
English (GB): Keuleers (2011)	Orthographic C	0.313227	0.313078	150,150.2	150,187.9	13,857	11.19352	0.000823	TRUE	LD_RT
English (GB): Keuleers (2011)	Base (Length + Frequency)	0.205377	0.205263	– 15,059.3	– 15,029.1	13,863				LD_ACC
English (GB): Keuleers (2011)	OND	0.211217	0.211046	– 15,159.5	– 15,121.9	13,863	102.6005	4.97E-24	TRUE	LD_ACC
English (GB): Keuleers (2011)	OLD20	0.210819	0.210648	– 15,152.5	– 15,114.9	13,863	95.56235	1.69E-22	TRUE	LD_ACC
English (GB): Keuleers (2011)	PLD20	0.206368	0.206196	– 15,074.6	– 15,036.9	13,863	17.3062	3.20E-05	TRUE	LD_ACC
English (GB): Keuleers (2011)	Orthographic C	0.205406	0.205234	– 15,057.8	– 15,020.1	13,863	0.507686	0.476154	FALSE	LD_ACC
English (US): Balota (2007)	Base (Length + Frequency)	0.387263	0.3872	223,023	223,054.5	19,465				LD_RT
English (US): Balota (2007)	OND	0.390755	0.390661	222,913.7	222,953.1	19,465	111.536	5.31E-26	TRUE	LD_RT
English (US): Balota (2007)	OLD20	0.404589	0.404498	222,466.6	222,506	19,465	566.3118	2.06E-123	TRUE	LD_RT
English (US): Balota (2007)	PLD20	0.40546	0.405369	222,438.1	222,477.5	19,465	595.6473	1.31E-129	TRUE	LD_RT
English (US): Balota (2007)	Orthographic C	0.387494	0.387399	223,017.6	223,057	19,465	7.331871	0.00678	TRUE	LD_RT
English (US): Balota (2007)	Base (Length + Frequency)	0.177407	0.177322	– 31,171.1	– 31,139.6	19,465				LD_ACC
English (US): Balota (2007)	OND	0.179017	0.17889	– 31,207.3	– 31,167.9	19,465	38.16366	6.63E-10	TRUE	LD_ACC
English (US): Balota (2007)	OLD20	0.187839	0.187714	– 31,417.6	– 31,378.2	19,465	249.9709	5.88E-56	TRUE	LD_ACC
English (US): Balota (2007)	PLD20	0.182112	0.181986	– 31,280.8	– 31,241.4	19,465	111.9581	4.30E-26	TRUE	LD_ACC
English (US): Balota (2007)	Orthographic C	0.177473	0.177347	– 31,170.7	– 31,131.3	19,465	1.573037	0.209782	FALSE	LD_ACC
Spanish: Aguasvivas (2018)	Base (Length + Frequency)	0.015651	0.015441	141,358.8	141,387.4	9368				LD_RT
Spanish: Aguasvivas (2018)	OND	0.021638	0.021324	141,303.6	141,339.4	9368	57.30095	4.09E-14	TRUE	LD_RT
Spanish: Aguasvivas (2018)	OLD20	0.015695	0.01538	141,360.4	141,396.1	9368	0.421986	0.515963	FALSE	LD_RT
Spanish: Aguasvivas (2018)	PLD20	0.015654	0.015339	141,360.8	141,396.5	9368	0.032072	0.857873	FALSE	LD_RT
Spanish: Aguasvivas (2018)	Orthographic C	0.015721	0.015405	141,360.1	141,395.9	9368	0.664025	0.415163	FALSE	LD_RT
Spanish: Aguasvivas (2018)	Base (Length + Frequency)	0.060416	0.060216	– 17,901.9	– 17,873.3	9368				LD_ACC
Spanish: Aguasvivas (2018)	OND	0.113558	0.113274	– 18,445.3	– 18,409.6	9368	561.3643	1.43E-120	TRUE	LD_ACC
Spanish: Aguasvivas (2018)	OLD20	0.060514	0.060213	– 17,900.8	– 17,865.1	9368	0.973976	0.323716	FALSE	LD_ACC
Spanish: Aguasvivas (2018)	PLD20	0.060453	0.060152	– 17,900.2	– 17,864.5	9368	0.370191	0.542915	FALSE	LD_ACC
Spanish: Aguasvivas (2018)	Orthographic C	0.061412	0.061111	– 17,909.8	– 17,874.1	9368	9.929565	0.001632	TRUE	LD_ACC
French: Ferrand (2011)	Base (Length + Frequency)	0.43889	0.437966	12,785.61	12,806.02	1217				LD_RT
French: Ferrand (2011)	OND	0.451778	0.450422	12,759.33	12,784.85	1217	28.51682	1.11E-07	TRUE	LD_RT
French: Ferrand (2011)	OLD20	0.447974	0.446609	12,767.74	12,793.26	1217	19.96096	8.64E-06	TRUE	LD_RT
French: Ferrand (2011)	PLD20	0.44226	0.44088	12,780.27	12,805.8	1217	7.328799	0.006881	TRUE	LD_RT
French: Ferrand (2011)	Orthographic C	0.439173	0.437786	12,786.99	12,812.51	1217	0.612945	0.433834	FALSE	LD_RT
French: Ferrand (2010)	Base (Length + Frequency)	0.223522	0.223446	229,353.6	229,385.3	20,524				LD_RT
French: Ferrand (2010)	OND	0.226562	0.226449	229,275	229,314.7	20,524	80.66157	2.90E-19	TRUE	LD_RT
French: Ferrand (2010)	OLD20	0.236859	0.236747	229,000	229,039.6	20,524	358.6058	2.69E-79	TRUE	LD_RT
French: Ferrand (2010)	PLD20	0.233352	0.23324	229,094.1	229,133.7	20,524	263.1185	8.33E-59	TRUE	LD_RT
French: Ferrand (2010)	Orthographic C	0.230294	0.230181	229,175.8	229,215.4	20,524	180.5364	5.52E-41	TRUE	LD_RT
Dutch: Brysbaert (2019)	Base (Length + Frequency)	0.156633	0.156502	156,156.8	156,186.6	12,851				LD_RT
Dutch: Brysbaert (2019)	OND	0.177889	0.177697	155,830.8	155,868.1	12,851	332.1615	2.72E-73	TRUE	LD_RT
Dutch: Brysbaert (2019)	OLD20	0.163692	0.163497	156,050.8	156,088.1	12,851	108.4336	2.72E-25	TRUE	LD_RT
Dutch: Brysbaert (2019)	PLD20	0.16588	0.165686	156,017.1	156,054.4	12,851	142.4251	1.17E-32	TRUE	LD_RT
Dutch: Brysbaert (2019)	Orthographic C	0.157717	0.157521	156,142.3	156,179.6	12,851	16.53659	4.80E-05	TRUE	LD_RT
Dutch: Brysbaert (2019)	Base (Length + Frequency)	0.084793	0.084651	– 47,324	– 47,294.2	12,851				LD_ACC
Dutch: Brysbaert (2019)	OND	0.094618	0.094407	– 47,460.7	– 47,423.4	12,851	139.4149	5.23E-32	TRUE	LD_ACC
Dutch: Brysbaert (2019)	OLD20	0.088759	0.088547	– 47,377.8	– 47,340.5	12,851	55.91657	8.05E-14	TRUE	LD_ACC
Dutch: Brysbaert (2019)	PLD20	0.088695	0.088482	– 47,376.9	– 47,339.6	12,851	55.00361	1.28E-13	TRUE	LD_ACC
Dutch: Brysbaert (2019)	Orthographic C	0.084868	0.084654	– 47,323.1	– 47,285.8	12,851	1.049237	0.305702	FALSE	LD_ACC

*R*^2^ = R squared; Adj. *R*^2^ = adjusted *R*^2^; AIC = Akaike information criterion; BIC = Bayesian information criterion; Obs. = number of words in the dataset; F: The F-statistic; Sig.: Indicates whether the model significantly differs from the baseline (TRUE/FALSE) based on the *p* value; DV = dependent variable; LD_RT = lexical decision reaction time; LD_ACC = lexical decision accuracy

## Appendix 5

**Table 7:** Multiple regression model results for naming data

Dataset	Model	*R* ^2^	Adj. *R*^2^	AIC	BIC	Obs	*F*	*p*	Sig	DV
English (US): Balota (2007)	Base (Length + Frequency)	0.323937	0.323867	215,406.3	215,437.8	19,465				NMG_RT
English (US): Balota (2007)	OND	0.327479	0.32734	215,308.1	215,355.3	19,465	51.24012	6.38E-23	TRUE	NMG_RT
English (US): Balota (2007)	OLD20	0.331779	0.331641	215,183.2	215,230.5	19,465	114.1825	5.01E-50	TRUE	NMG_RT
English (US): Balota (2007)	PLD20	0.344001	0.343866	214,823.9	214,871.1	19,465	297.6017	4.90E-128	TRUE	NMG_RT
English (US): Balota (2007)	Orthographic C	0.325905	0.325767	215,353.5	215,400.8	19,465	28.41454	4.76E-13	TRUE	NMG_RT
English (US): Balota (2007)	Base (Length + Frequency)	0.055265	0.055168	– 57,388.1	– 57,356.6	19,465				NMG_ACC
English (US): Balota (2007)	OND	0.055829	0.055635	– 57,395.7	– 57,348.5	19,465	5.813325	0.002993	TRUE	NMG_ACC
English (US): Balota (2007)	OLD20	0.063843	0.06365	– 57,561.6	– 57,514.4	19,465	89.15596	2.86E-39	TRUE	NMG_ACC
English (US): Balota (2007)	PLD20	0.070359	0.070168	– 57,697.6	– 57,650.3	19,465	157.9836	8.70E-69	TRUE	NMG_ACC
English (US): Balota (2007)	Orthographic C	0.055995	0.055801	– 57,399.1	– 57,351.9	19,465	7.524528	0.000541	TRUE	NMG_ACC
French: Ferrand (2011)	Base (Length + Frequency)	0.082915	0.081403	12,158.2	12,178.62	1216				NMG_RT
French: Ferrand (2011)	OND	0.084464	0.08144	12,160.15	12,190.77	1216	1.024295	0.35936	FALSE	NMG_RT
French: Ferrand (2011)	OLD20	0.08526	0.082239	12,159.09	12,189.71	1216	1.552459	0.212148	FALSE	NMG_RT
French: Ferrand (2011)	PLD20	0.084027	0.081002	12,160.73	12,191.35	1216	0.735339	0.479557	FALSE	NMG_RT
French: Ferrand (2011)	Orthographic C	0.085851	0.082831	12,158.3	12,188.92	1216	1.944559	0.143497	FALSE	NMG_RT

*R*^2^ = R squared; Adj. *R*^2^ = adjusted *R*^2^; AIC = Akaike information criterion; BIC = Bayesian information criterion; Obs. = number of words in the dataset; F: The F-statistic; Sig.: Indicates whether the model significantly differs from the baseline (TRUE/FALSE) based on the *p* value; DV = dependent variable; NMG_RT = naming reaction time; NMG_ACC = naming accuracy

## Footnotes

1 The English language has two corpora in our tool: one for US English, and the other for GB English.

2 https://github.com/stopwords-iso/stopwords-iso

3 https://github.com/GNUAspell/aspell

4 https://github.com/hunspell/hunspell

5 https://github.com/languagetool-org/languagetool

6 https://github.com/bakwc/JamSpell

7 https://github.com/espeak-ng/espeak-ng

8 https://github.com/espeak-ng/espeak-ng/blob/master/docs/languages.md

9 https://github.com/AlaaAlzahrani/Jiwar_database

10 https://jiwar.readthedocs.io/en/latest/languages/supported.html#languages-with-built-in-corpora

11 https://jiwar.readthedocs.io/en/latest/index.html

12 The appendix contains results for two correlation methods: Pearson's r and Spearman's rho, along with their 95% confidence intervals. In the correlation plots, we display only Spearman's rho values. We plotted Spearman's correlation because: (1) the two methods produced similar results, and (2) Spearman's is more robust since it can handle outliers and nonlinear relationships (Kowalski, 1972; Schober et al., 2018).

13 https://jiwar.readthedocs.io/en/latest/index.html

## Code availability

All R scripts used to analyze the data are available at OSF: https://osf.io/psrav/?view_only=1cde0e02e3aa47189fa5764aac3916da. All Python code scripts are publicly available on GitHub: https://github.com/AlaaAlzahrani/Jiwar.

## Declarations

### Open Practices Statement

The data and materials for this study are available at OSF (https://osf.io/psrav/?view_only=1cde0e02e3aa47189fa5764aac3916da) and GitHub (https://github.com/AlaaAlzahrani/Jiwar). The study was not preregistered.
